# Quantification of Blood Viscoelasticity under Microcapillary Blood Flow

**DOI:** 10.3390/mi14040814

**Published:** 2023-04-03

**Authors:** Yang Jun Kang

**Affiliations:** Department of Mechanical Engineering, Chosun University, 309 Pilmun-daero, Dong-gu, Gwangju 61452, Republic of Korea; yjkang2011@chosun.ac.kr; Tel.: +82-62-230-7052; Fax: +82-62-230-7055

**Keywords:** blood viscoelasticity, two compliance coefficients, coflowing method, discrete fluidic circuit modeling, nonlinear curve fitting

## Abstract

Blood elasticity is quantified using a single compliance model by analyzing pulsatile blood flow. However, one compliance coefficient is influenced substantially by the microfluidic system (i.e., soft microfluidic channels and flexible tubing). The novelty of the present method comes from the assessment of two distinct compliance coefficients, one for the sample and one for the microfluidic system. With two compliance coefficients, the viscoelasticity measurement can be disentangled from the influence of the measurement device. In this study, a coflowing microfluidic channel was used to estimate blood viscoelasticity. Two compliance coefficients were suggested to denote the effects of the polydimethylsiloxane (PDMS) channel and flexible tubing (*C*_1_), as well as those of the RBC (red blood cell) elasticity (*C*_2_), in a microfluidic system. On the basis of the fluidic circuit modeling technique, a governing equation for the interface in the coflowing was derived, and its analytical solution was obtained by solving the second-order differential equation. Using the analytic solution, two compliance coefficients were obtained via a nonlinear curve fitting technique. According to the experimental results, *C*_2_/*C*_1_ is estimated to be approximately 10.9–20.4 with respect to channel depth (*h* = 4, 10, and 20 µm). The PDMS channel depth contributed simultaneously to the increase in the two compliance coefficients, whereas the outlet tubing caused a decrease in *C*_1_. The two compliance coefficients and blood viscosity varied substantially with respect to homogeneous hardened RBCs or heterogeneous hardened RBCs. In conclusion, the proposed method can be used to effectively detect changes in blood or microfluidic systems. In future studies, the present method can contribute to the detection of subpopulations of RBCs in the patient’s blood.

## 1. Introduction

The rheological properties of blood are significantly influenced by highly flexible red blood cells (RBCs) and aqueous plasma. Physiological disorders (i.e., hypertension [1,2], diabetes [3,4], sickle cell anemia [5,6], and malaria [7,8]) contribute to altered hemorheological properties (i.e., hematocrit (Hct) [9,10], RBC deformability, RBC aggregation, and viscosity) [11,12]. The mechanical properties of blood are regarded as potential biomarkers and have been widely used in clinical settings [13,14]. Among the mechanical properties of blood, fluid viscosity is commonly used to monitor substantial variations in blood. Blood behaves as a viscoelastic fluid (i.e., viscosity or elasticity) [15]. Blood viscoelasticity is generally obtained with respect to sinusoidally varying angular velocities. In other words, a conventional rheometer (i.e., cone-and-plate or plate-and-plate), as gold standard, can provide quantitative information about blood viscoelasticity [16,17]. However, the conventional method requires a large blood volume (on the order of milliliters) to fill the space between the two plates, tedious cleaning procedures per experiment, and an expert for confirming reliable and consistent data, in addition to having a high operating cost. Additionally, owing to its bulky size and precision requirements, the conventional rheometer is available in limited research environments [18].

A microfluidic platform is a potential solution for resolving the problems associated with the conventional rheometer. Microfluidic devices offer distinctive merits, including low blood volume consumption, short measurement, and high sensitivity. Most importantly, the microfluidic device embodies a bio-mimicking interface or environment (i.e., pressure-driven flow, similar dimensions, and configuration) when compared with in vivo capillary blood flow. Therefore, microfluidic devices have been suggested and adapted to obtain rheological properties [19,20]. First, to measure the blood viscosity under a constant shearing flow, the flow rate is set to a constant value using a syringe pump or pressure source. Using the Hagen–Poiseuille equation (i.e., pressure drop = fluidic resistance × flow rate), the blood viscosity is obtained by quantifying the pressure drop or fluidic resistance under constant shearing blood flow. According to previous studies, several quantification methods, including droplet velocity [21], droplet length [22,23], blood advancing velocity [24,25,26], digital flow compartment [27,28], and parallel flow [29,30], have been demonstrated to effectively measure fluidic resistance (or viscosity) in microfluidic channels. According to the parallel flow method [29,31], the reference fluid (i.e., 1× phosphate-buffered saline [PBS]) and test fluid (i.e., blood) are supplied into each inlet at the same flow rate, and the viscosity of the reference fluid is specified in advance. As the interface is determined by the viscosity ratio of the two fluids, the blood viscosity is obtained by detecting the interface without calibration. Next, to obtain blood viscoelasticity, the flow rate is set to a periodic on/off or sinusoidal pattern. Under transient blood flow, blood velocity [32] or image intensity [33] gradually decreases over time. On the basis of the transient behaviors of physical parameters (i.e., velocity and image intensity [34]), the regression formula was assumed to be *I* (*t*) = *I*_0_ + *I*_1_ exp (−*t/κ*) [33]. Using temporal variations in image intensity, time constant *κ* was obtained by conducting nonlinear regression analysis. Using the linear Maxwell model (i.e., time constant = viscosity/elasticity) [13], blood elasticity is obtained by dividing viscosity by the time constant (i.e., elasticity = viscosity/time constant) [35]. Here, the blood viscosity should be specified in advance to extract blood elasticity from the time constant. The Maxwell model was suggested to represent the viscoelasticity of a single RBC under Couette flow conditions. However, for blood flow in a microfluidic channel, a new mathematical model is required to effectively evaluate the blood elasticity effect. Recently, our group suggested that a single compliance element should be adopted to extract blood elasticity under transient blood flows [36]. Compliance has a reciprocal relationship with elasticity (i.e., compliance ∝ 1/elasticity) [37]. Although it is aimed at obtaining blood elasticity, a single compliance element includes the combined effects of several factors including RBC elasticity, flexible microfluidic channels, and polyethylene tubing. Therefore, it is necessary to update the previous mathematical model. Therefore, instead of a single compliance element, two compliance elements are suggested to effectively separate the contributions of RBC elasticity from the other two factors.

In this study, two compliance elements are added to effectively evaluate the contribution of the RBC elastic effect. As the coflowing method gives accurate blood viscosity without calibration, the present study adopts a coflowing microfluidic channel. On the basis of the discrete fluidic circuit modeling technique, a new governing equation for the microfluidic system is derived as a second-order differential equation. The interfacial location is selected as the dependent variable and used for obtaining blood viscosity and two compliances. According to the governing equation, the blood viscosity is obtained using the steady values of the interface under steady blood flow. Next, nonlinear regression analysis is conducted to obtain two compliance elements after transient variations of the interface are obtained under transient blood flow. The two compliance elements exhibit individual contributions of RBC elasticity, microfluidic device, and tubing.

Compared with previous methods [35,36], the elastic effect of the microfluidic system is modeled as two compliance elements rather than a single compliance element. The governing equation of the microfluidic system is changed from the first-order differential equation to a second-order differential equation. The two compliance elements are effectively used to evaluate the elastic effect of RBCs, flexible device, and tubing. As a demonstration, blood viscosity and two compliance coefficients are used to detect homogeneous and heterogeneous hardened RBCs.

## 2. Materials and Methods

### 2.1. Microfluidic Device and Experimental Procedures

**Figure 1:**
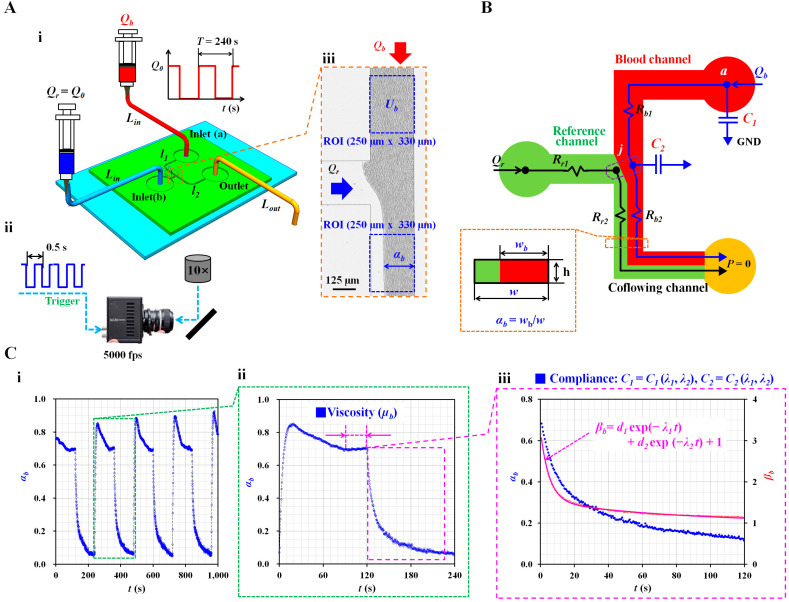
Proposed method for measurement of blood viscoelasticity under microcapillary blood flow. (**A**) Schematic diagram of experimental setup, including a microfluidic device, two syringe pumps, and image acquisition system. (**i**) Microfluidic device consisting of two inlets (a, b), an outlet, and microfluidic channels (i.e., reference channel, blood channel, and coflowing channel). Using two syringe pumps, blood flow rate (*Q_b_*) set to a periodic on/off profile (i.e., amplitude: *Q*_0_, and period: *T*). Flow rate of reference fluid set to a constant flow rate (i.e., *Q_r_* = *Q*_0_). The corresponding length of each polyethylene tubing is denoted as *L_in_* (for inlet tubing) and *L_out_* (for outlet tubing). (**ii**) Image acquisition system for capturing blood flow in the microfluidic channels. With high-speed camera set to 5000 fps, microscopic images are sequentially captured at an interval of 0.5 s. (**iii**) Microscopic image showing interface (*α_b_*) in the coflowing channel and blood velocity (*U_b_*) in the blood channel. (**B**) Mathematical representation with discrete fluidic circuit elements for estimating viscoelasticity. The microfluidic system (i.e., two fluids, flexible microfluidic device, and polyethylene tubing) is modeled as discrete fluidic elements, including flow rate (*Q_r_* and *Q_b_*), fluidic resistance (*R_r_*_1_, *R_r_*_2_, *R_b_*_1_, and *R_b_*_2_), and compliance (*C*_1_ and *C*_2_). Here, ‘►’ denotes the zero value of pressure (*P* = 0). (**C**) As a preliminary demonstration, control blood (Hct = 50%) was prepared by adding normal RBCs to autologous plasma, with flow rate of blood set to on/off pattern (i.e., *Q*_0_ = 1 mL/h, *T* = 240 s), and flow rate of reference fluid set to *Q_r_* = 1 mL/h. (**i**) Temporal variations of *α_b_* for up to 1000 s. (**ii**) Quantification of blood viscosity using steady variations of *α_b_* after turning on syringe pump. (**iii**) Temporal variations of *α_b_* and *β_b_* = (1 − *α_b_*)^−1^ after turning off syringe pump. Two compliances were obtained by conducting nonlinear regression of transient behaviors of *β_b_* (i.e., *β_b_* = *d*_1_ exp [−*λ*_1_*t*] + *d*_2_ exp [−*λ*_2_*t*] +1).

To measure the blood viscosity and two compliance coefficients, a microfluidic device was designed with two inlets (a and b), an outlet, and three microfluidic channels (i.e., reference, blood, and coflowing channels), as shown in Figure 1(A-i). The corresponding lengths of the blood and coflowing channels were set to *l*_1_ = 7500 µm and *l*_2_ = 4800 µm. The three channels were set to have the same width (*w* = 250 µm). However, to evaluate the effect of the channel depth on the compliance effect, the three channel depths were set to *h* = 4, 10, and 20 μm.

A four-inch silicon master mold was fabricated using conventional microelectromechanical system fabrication techniques (i.e., photolithography and deep reactive ion etching). To perform soft lithography on the silicon master mold, polydimethylsiloxane (PDMS) (Sylgard 184, Dow Corning, Midland, Michigan, USA) was mixed with a curing agent in a mass ratio of 10:1 (i.e., PMDS to curing agent). The PDMS mixture was poured onto a silicon master mold adhered to a petri dish. Air bubbles in the PDMS were removed using a vacuum pump for 1 h. After curing the PDMS mixture in a convection oven at 70 °C for 1 h, the PDMS block was peeled off from the silicon master mold. The PDMS device was then cut using a razor blade, and three ports (two inlets and one outlet) were punched with a biopsy punch (outer diameter = 0.75 mm). After oxygen plasma treatment (CUTE-MPR, Femto Science Co., Gyeonggi-do, Korea), a microfluidic device was fabricated by bonding a PDMS device on a glass substrate. To ensure strong bonding between the two surfaces, the device was placed on a hot plate (120 °C) for 10 min.

Two types of inlet tubing (inner diameter = 250 µm, *L_in_* = 300 mm) were attached to each inlet port (a, b). The outlet tubing (inner diameter = 250 µm, *L_ou_*_t_ = 200–400 mm) was connected to the outlet port. To remove the initial air in the microfluidic channels and avoid nonspecific binding of plasma protein to the surface of the microfluidic channels, bovine serum albumin (2 mg/mL) was loaded from the outlet. After 10 min, blood (approximately 0.3 mL) and reference fluid (approximately 0.3 mL) were loaded into two disposable syringes (1 mL). The needle in each syringe was connected to the end of each inlet tube. Thereafter, two syringes were installed in each syringe pump (NeMESYS; Cetoni GmbH, Germany). Blood was supplied to inlet (a) in a periodic on/off pattern (amplitude: *Q*_0_ and period: *T* = 240 s). The reference fluid was supplied to inlet (b) at a constant flow rate (*Q*_0_).

As shown in Figure 1(A-ii), the microfluidic device was positioned on an optical microscope (BX51, Olympus, Japan) equipped with a 10× objective lens (NA = 0.25). A high-speed camera (FASTCAM MINI, Photron, London, United Kingdom) was used to capture the blood flow images in the microfluidic channels. To clearly visualize blood flow, the frame rate was set to 5000 fps (frame per second). Using a function generator (WF1944B, NF Corporation, Yokohama, Japan), a pulse signal with a period of 0.5 s triggered the high-speed camera. Two microscopic images were captured sequentially at intervals of 0.5 s.

### 2.2. Quantification of Interface in the Coflowing Channel

In Figure 1(A-iii), *Q_r_* and *Q_b_* represent the flow rate of the reference fluid in the reference channel and blood flow rate in the blood channel, respectively. To obtain the interface in the coflowing channel, grayscale images were converted into binary-scale images using Otsu method [38]. A specific region of interest (ROI, 250 µm × 330 µm) was selected within the coflowing channel. By averaging the blood-filled widths distributed within the ROI, the average blood-filled width was calculated and denoted by *w_b_*. Dividing the averaged blood-filled width (*w_b_*) by the channel width (*w*), the dimensionless form of the interface could be expressed as *α_b_* = *w_b_*/*w*. The variations in the interface were then obtained at intervals of 0.5 s.

### 2.3. Quantification of Blood Velocity in the Blood Channel

To monitor blood flow in the blood channel supplied from a syringe pump (*Q_b_*), a specific ROI (250 µm × 330 µm) was selected at a far distance from the junction (‘*j*’). Blood velocity fields were obtained with time-resolved microparticle image velocimetry [39]. The interrogation window was set to 32 × 32 pixels. One pixel corresponded to 1.67 µm. The window overlap set to 50%. The blood velocity (*U_b_*) was then obtained by arithmetically averaging the velocity fields distributed over the ROI.

### 2.4. Mathematical Representation of the Present Microfluidic System

To quantify the blood viscosity and two compliance coefficients, the blood flow rate was set to a periodic on/off pattern. By analyzing the interface (*α_b_*) in the coflowing channel, the viscosity and two compliance coefficients were obtained at constant blood flow and transient blood flow, respectively. Three assumptions were made to derive the governing differential equation of blood flow in the microfluidic system. First, by designing a microfluidic channel with a low aspect ratio (AR) (AR = *h*/*w* < 0.1), the fluid flow in the rectangular channel was approximated as a two-dimensional distribution. Second, by supplying it at a sufficiently high shear rate of $\dot{\gamma }$ > 10^3^ s^−1^, blood was assumed to be a Newtonian fluid. Lastly, referring to a previous study [35], the blood viscosity remained constant within a specific interface (i.e., 0.1 < *α_b_* < 0.9). Therefore, the present study did not consider viscosity reduction owing to the cell-free layer (i.e., Fåhraeus effect). According to previous studies [35,36,40], the contribution of blood elasticity is represented by a single compliance element. By suddenly stopping the blood flow, the characteristic time was quantified by analyzing the transient behaviors of the cell-to-liquid interface in the coflowing channel. Next, according to the linear Maxwell model (i.e., characteristic time = viscosity/elasticity), the blood elasticity was obtained by dividing the viscosity by the characteristic time. However, as a limitation of the previous study, the single compliance element represented the combined effects of blood elasticity, tubing effect, and PDMS device effect. Two compliance elements were added to the fluidic circuit model to separate the individual contributions of the three components. As shown in Figure 1B, the first compliance element (*C*_1_) represents the compliance of the inlet tubing and PDMS channel, while the second compliance element (*C*_2_) denotes that of blood elasticity. As expected, blood exhibited a higher value of compliance than the tubing or device; therefore, it was assumed that *C*_1_ is smaller than *C*_2_ (*C*_1_ < *C*_2_). Owing to the two compliance elements, the order of the governing differential equation increased from first-order to second-order. As a result, the governing equation became more complex. Under a transient blood flow, the analytical solution of *α_b_* was derived by solving the governing equation. Thereafter, two compliance coefficients were obtained by conducting a nonlinear regression analysis.

As shown in Figure 1B, the microfluidic system (i.e., two fluids, a flexible microfluidic device, and polyethylene tubing) was modeled as discrete fluidic elements, including flow rate elements (*Q_r_* and *Q_b_*), fluidic resistance elements (*R_r_*_1_, *R_r_*_2_, *R_b_*_1_, and *R_b_*_2_), and compliance elements (*C*_1_ and *C*_2_). The subscripts ‘*r*’ and ‘*b*’ represent the reference fluid and blood, respectively; symbol ‘►’ denotes the zero value of pressure (*P* = 0), called ‘GND’. To model the coflowing channel filled with blood and reference fluid, the interface was assumed to be a virtual wall. Two fluid streams were modeled as two fluidic resistances (*R_r_*_2_ and *R_b_*_2_) connected in parallel. Subsequently, a correction factor (CF) was used to compensate for the difference between the physical model and simple mathematical model [35,41,42]. According to previous studies, the correction factor can be expressed as an interface (i.e., CF = CF [*α_b_*]). The fluidic resistance equations of two fluid streams (i.e., width of the reference fluid stream = [1 − *α_b_*] × *w*, and width of blood stream = *α_b_* × *w*) in the coflowing channel were derived as

(1) $$R_{r2}=\frac{12\mu_{r}l_{2}}{\left(1-\alpha_{b}\right)wh^{3}},$$

(2) $$R_{b2}=\frac{12\mu_{b}l_{2}}{{CF\alpha }_{b}wh^{3}},$$

where $\mu_{r}\;\mathrm{and}\;\mu_{b}$ represent the viscosities of the reference fluid and blood, respectively. Next, the fluidic resistance equation for the upper channel filled with blood (i.e., channel length = *l*_1_) was derived as

(3) $$R_{b1}=\frac{12\mu_{b}l_{1}}{wh^{3}}.$$

As a function of the junction point (‘*j*’) where both channels were joined, mass conservation of the reference fluid resulted in the following expression:

(4) $$Q_{r}=\frac{P_{j}}{R_{r2}}.$$

In addition, as a function of each point (i.e., ‘*a’* and ’*j*’), the mass conservation of blood yields the following equations:

(5) $$Q_{b}=C_{1}\frac{d}{dt}P_{a}+\frac{P_{a}-P_{j}}{R_{b1}},$$

(6) $$\frac{P_{a}-P_{j}}{R_{b1}}=C_{2}\frac{d}{dt}P_{j}+\frac{P_{j}}{R_{b2}}.$$

When Equations (1)–(4) and (6) were substituted into Equation (5), the differential equation of the interface was derived as

(7) $$\left(R_{b1}R_{r3}C_{1}C_{2}\right)\frac{d^{2}}{dt^{2}}\left(\frac{1}{1-\alpha_{b}}\right)+\left(C_{1}R_{r3}+C_{2}R_{r3}\right)\frac{d}{dt}\left(\frac{1}{1-\alpha_{b}}\right)\;+\left(R_{r3}C_{1}\frac{l_{1}}{l_{2}}\right)\frac{d}{dt}\left(\frac{\alpha_{b}CF}{1-\alpha_{b}}\right)+\left(\frac{\mu_{r}}{\mu_{b}}\right)\left(\frac{\alpha_{b}CF}{1-\alpha_{b}}\right)=\frac{Q_{b}}{Q_{r}},$$

where $R_{r3}=\frac{12\mu_{r}l_{2}}{wh^{3}}$. Under steady blood flow conditions, the equation for blood viscosity was derived as

(8) $$\mu_{b}=\mu_{r}\times \left(\frac{\alpha_{b}CF}{1-\alpha_{b}}\right)\times \left(\frac{Q_{r}}{Q_{b}}\right).$$

Next, by setting the blood flow rate to zero (*Q_b_* = 0), the right-hand term of Equation (7) becomes equal to zero (*Q_b_*/*Q_r_* = 0). However, the governing equation includes the nonlinear term $\frac{\alpha_{b}CF(\alpha_{b})}{1-\alpha_{b}}$. Therefore, it is necessary to convert the nonlinear term into a linear term to obtain an approximate solution for the transient blood flow. CF (*α_b_*) was assumed as a weighting function in the expression of $\frac{\alpha_{b}CF(\alpha_{b})}{1-\alpha_{b}}$. That is, the CF (*α_b_*) was varied continuously within the specific range of the interface. For convenience, it was assumed that CF (*α_b_*) remained the constant over interface. Using an arithmetic averaging procedure, the constant CF_0_ was obtained by averaging CF (*α_b_*) × *α_b_*/(1 − *α_b_*) within a specific value of interface. According to the approximation procedure reported in a previous study [43],

(9) $$\sum \frac{\alpha_{b}CF\left(\alpha_{b}\right)}{1-\alpha_{b}}\approx \sum \frac{\alpha_{b}}{1-\alpha_{b}}\times {CF}_{0},$$

where the term $\frac{\alpha_{b}CF(\alpha_{b})}{1-\alpha_{b}}$ is simplified as

(10) $$\frac{\alpha_{b}CF(\alpha_{b})}{1-\alpha_{b}}\approx \frac{\alpha_{b}{CF}_{0}}{1-\alpha_{b}}.$$

Under transient blood flow conditions, Equation (7) becomes the following second-order linear differential equation:

(11) $$a\frac{d^{2}}{dt^{2}}\left(\beta_{b}\right)+b\frac{d}{dt}\left(\beta_{b}\right)+c\beta_{b}=c,$$

where $\beta_{b}$ can be expressed as $\beta_{b}={\left(1-\alpha_{b}\right)}^{-1}$. The three constants are given as

$$a=R_{b1}R_{r3}C_{1}C_{2},\;\;\;\;\;\;\;b=R_{r3}\left(C_{1}{+C}_{2}+C_{1}\times \frac{l_{1}}{l_{2}}{\times CF}_{0}\right),\;c=\left(\frac{\mu_{r}}{\mu_{b}}\right)\times {CF}_{0}.$$

The general solution to the differential Equation (11) was derived as

(12) $$\beta_{b}=d_{1}\exp\left(-\lambda_{1}t\right)+d_{2}\exp\left(-\lambda_{2}t\right)+1,$$

where the two eigenvalues (*λ*_1_ and *λ*_2_) satisfy the following relations:

(13) $$\lambda_{1}+\lambda_{2}=\frac{C_{1}{+C}_{2}+C_{1}\times \frac{l_{1}}{l_{2}}{\times CF}_{0}}{R_{b1}C_{1}C_{2}},$$

(14) $$\lambda_{1}\times \lambda_{2}=\frac{1}{R_{b1}{R_{r3}C}_{1}C_{2}}\times \left(\frac{\mu_{r}}{\mu_{b}}\right)\times {CF}_{0}.$$

In this study, the two eigenvalues (*λ*_1_ and *λ*_2_) were obtained by conducting a nonlinear regression analysis of the temporal variations of $\beta_{b}={\left(1-\alpha_{b}\right)}^{-1}$. As a preliminary demonstration (shown in Figure 1(C-i)), variations in the interface (*α_b_*) were obtained under periodic on/off blood flow. Control blood (Hct = 50%) was prepared by adding normal RBCs to the autologous plasma. The blood flow rate was set to a pulsatile pattern (*Q*_0_ = 1 mL/h and *T* = 240 s), and the flow rate of the reference fluid was set at *Q_r_* = 1 mL/h. After turning on a syringe pump, interface arrived at constant value after an elapse of 70 s. To obtain blood viscosity under constant shearing blood flow, it was necessary to guarantee enough interval of constant interface over time. According to a transient response, the turn-on time of the syringe pump was set to 120 s, while the turn-off time of the syringe pump was fixed at 120 s. As shown in Figure 1(C-ii), using Equation (8), the blood viscosity (*µ_b_*) could be obtained with steady values of *α_b_*. Next, as shown in Figure 1(C-iii), the temporal variations in *α_b_* were converted into *β_b_* = (1 − *α_b_*)^−1^. Subsequently, the two eigenvalues (*λ*_1_ and *λ*_2_) were calculated as *λ*_1_ = 0.1945 s^−1^ and λ_2_ = 0.0127 s^−1^ by conducting a nonlinear regression of *β_b_* (i.e., *β_b_* = *d*_1_ exp [−*λ*_1_*t*] + *d*_2_ exp [−*λ*_2_*t*] +1). The two compliance coefficients (*C*_1_ and *C*_2_) were obtained as *C*_1_ = 53.3 µm^3^/mPa and *C*_2_ = 1398.2 µm^3^/mPa by simultaneously solving Equations (13) and (14).

### 2.5. Blood Preparation for Validating the Present Method

This study was conducted in accordance with the principles of the Declaration of Helsinki. The protocol was approved by the ethics committee of Chosun University under the reference code (2-1041055-AB-N-01-2022-47). Packed red blood cell (RBC) bags (approximately 320 mL) and fresh frozen plasma (FFP) bags (approximately 320 mL) were purchased from the Gwangju–Chonnam blood bank (Gwangju, Korea). Before the blood test, RBCs and FFP were stored at 4 °C and −20 °C, respectively, in a refrigerator. To collect normal RBCs from concentrated RBCs, suspended blood was prepared by adding concentrated RBCs to 1× PBS (pH 7.4, Gibco, Life Technologies, California, USA). After operation of the centrifugal separator for approximately 10 min, the suspended blood was separated into two layers (i.e., upper and lower layers). Normal RBCs in the lower layer were retained by removing the upper layer (i.e., buffy layer and 1× PBS) from the suspended blood. The washing procedure was repeated thrice. After FFP was melted at a constant temperature of 25 °C, autologous plasma was prepared by removing debris from FFP using a syringe filter (mesh size = 5 μm, Minisart, Sartorius, Göttingen, Germany). To simulate various values of blood viscoelasticity, suspended blood was prepared by adding normal RBCs or hardened RBCs to specific diluents (i.e., 1× PBS and autologous plasma). To sufficiently rigidify normal RBCs, five different types of diluted glutaraldehyde (GA) (*C_GA_* = 2, 4, 6, 8, and 10 µL/mL) were prepared by adding GA (Grade II, 25% in H_2_O, Sigma-Aldrich, St. Louis, MO, USA) to 1× PBS. Normal RBCs were hardened by mixing them with each concentration of the GA solution for 10 min. After preparation of the suspended blood, specific experiments were conducted immediately to obtain consistent results.

## 3. Results and Discussion

### 3.1. Contribution of Channel Depth to Blood Viscosity under Steady Flow

**Figure 2:**
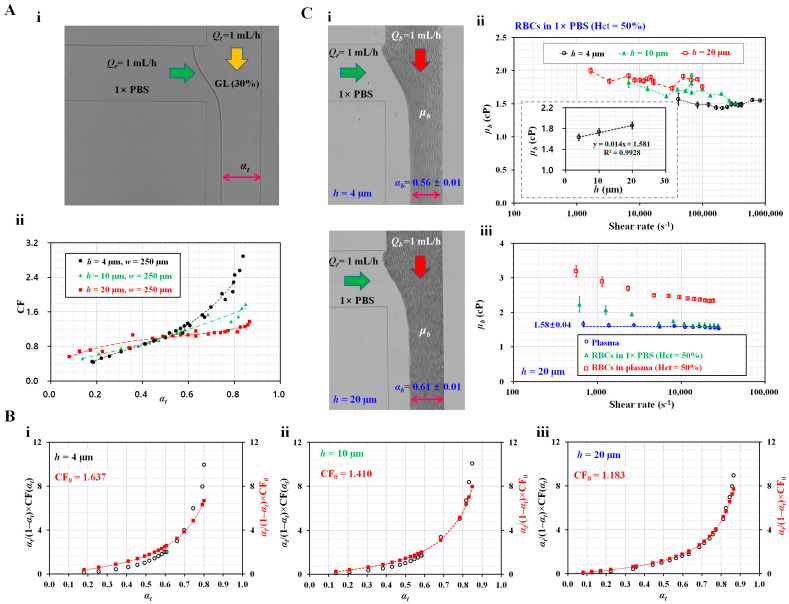
Contribution of channel depth (*h*) to blood viscosity under steady flow. (**A**) Quantification of correction factor with respect to channel depth. (**i**) Microscopic image showing interface of two fluids (i.e., test fluid: glycerin solution [30%], reference fluid: 1× PBS) in the coflowing channel, at the same flow rate (i.e., *Q_r_* = *Q_b_* = 1 mL/h); *α_t_* denotes the interface. (**ii**) Variations of correction factor (CF) with respect to channel depth (*h* = 4, 10, and 20 µm) and interface (*α_t_*). (**B**) Approximation of *α_t_*/(1 − *α_t_*) × CF (*α_t_*) as *α_t_*/(1 − *α_t_*) × CF_0_ with respect to channel depth (*h*) ([**i**] CF_0_ = 1.637 for *h* = 4 µm, [**ii**] CF_0_ = 1.41 for *h* = 10 µm, and [**iii**] CF_0_ = 1.183 for *h* = 20 µm). (**C**) Variations of blood viscosity (*µ_b_*) with respect to channel width. (**i**) Microscopic images showing interface with respect to *h* = 4 and 20 µm. (**ii**) Contribution to channel depth to *µ_b_*. Blood (Hct = 50%) was prepared by adding normal RBCs into 1× PBS. The inset shows variations of *µ_b_* with respect to *h*. (**iii**) Variations in viscosity of blood (plasma, normal RBCs in plasma, and normal RBCs in 1× PBS) with respect to shear rate. Here, channel depth was set to 20 µm. Hematocrit was fixed to 50%.

First, to measure the blood viscosity using Equation (8), it was necessary to obtain a correction factor within a specific range of the interface. Figure 2(A-i) shows a microscopic image of the interface of the two fluids (i.e., test fluid: glycerin [30%], reference fluid: 1× PBS) in the coflowing channel at the same flow rate (i.e., *Q_r_* = *Q_t_* = 1 mL/h); *α_t_* denotes the interface in the coflowing channel. Various values of *α_t_* were obtained by adjusting *Q_r_* or *Q_t_* using a syringe pump. By manipulating Equation (8), the correction factor (CF = CF [*α_t_*]) could be expressed as

(15) $$CF=\left(\frac{\mu_{t}}{\mu_{r}}\right)\times \left(\frac{1-\alpha_{t}}{\alpha_{t}}\right)\times \left(\frac{Q_{t}}{Q_{r}}\right),$$

where the viscosity of glycerin (30%) and 1× PBS was taken as *µ_t_* = 3 cP and *µ_r_* = 1 cP [44]. Using three channel depths (*h* = 4, 10, and 20 µm), variations in the interface were obtained by adjusting either *Q_r_* or *Q_t_* with a syringe pump. By substituting five parameters (*µ_t_*, *µ_r_*, *α_t_*, *Q_b_*, and *Q_r_*) into Equation (15), the correction factor was obtained as a function of the interface. Figure 2(A-ii) shows the variations in CF with respect to the channel depth (*h*) and interface. According to the linear regression analysis, the corresponding correction factor for each channel depth was obtained as a polynomial expression: (i) CF = 7.5698*α_t_*^3^ − 7.669*α_t_*^2^ + 4.1983*α_t_* − 0.1289 (R^2^ = 0.9858) for *h* = 4 µm; (ii) CF = 0.6931*α_t_*^2^ + 0.9378*α_t_* + 0.38 (R^2^ = 0.9525) for *h* = 10 µm; (iii) CF = 27.95*α_t_*^5^ − 54.452*α_t_*^4^ + 36.954*α_t_*^3^ − 11.062*α_t_*^2^ + 2.5117*α_t_* + 0.4163 (R^2^ = 0.9354) for *h* = 20 µm.

Next, to approximate the nonlinear term ($\frac{\alpha_{b}CF[\alpha_{b}]}{1-\alpha_{b}}$) in Equation (7), the constant correction factor (CF_0_) was obtained using Equation (9). The corresponding CF_0_ for each channel depth was obtained as (i) CF_0_ = 1.637 for *h* = 4 µm, (ii) CF_0_ = 1.41 for *h* = 10 µm, and (iii) CF_0_ = 1.183 for *h* = 20 µm. Figure 2B shows the difference between the original expression ($\frac{\alpha_{t}CF[\alpha_{b}]}{1-\alpha_{t}}$) and approximation expression ($\frac{\alpha_{t}{CF}_{0}}{1-\alpha_{t}}$) with respect to the interface and channel depth (*h* = 4, 10, and 20 µm). The lower value of the channel depth exhibited a slight difference when compared with a high channel depth. For a higher channel depth (*h* = 20 µm), the difference between the original expression and approximation expression was much smaller with respect to the specific interface.

Lastly, the blood viscosity was measured with respect to the channel depth. Figure 2(C-i) shows the microscopic images of the interface with respect to *h* = 4 and 20 µm. Blood (Hct = 50%) was prepared by adding normal RBCs to 1× PBS. The corresponding interface for each channel was obtained as *α_b_* = 0.56 ± 0.01 for *h* = 4 µm and *α_b_* = 0.61 ± 0.01 for *h* = 20 µm. Figure 2(C-ii) shows the effect of channel depth on *µ_b_* with respect to shear rate. According to shear rate formula in the rectangular channel (i.e., $\dot{\gamma }=\frac{6Q_{b}}{wh^{2}}$), channel depth contributed to decreasing shear rate. Shear rate decreased substantially at higher values of channel depth. The corresponding blood viscosity for each channel depth was obtained as *µ_b_* = 1.63 ± 0.04 cP for *h* = 4 µm, *µ_b_* = 1.73 ± 0.11 cP for *h* = 10 µm, and *µ_b_* = 1.86 ± 0.06 cP for *h* = 20 µm. The inset shows variations of *µ_b_* with respect to *h*. The channel depth substantially contributed to the increase in blood viscosity. The results are in agreement with those of a previous study [35]. Furthermore, variations in blood viscosity were obtained with respect to shear rate. The channel depth was set as *h* = 20 µm, and three types of blood (i.e., plasma, normal RBCs in plasma, and normal RBCs in 1× PBS) were prepared to evaluate variations in blood viscosity with respect to shear rate. The flow rates of both the fluids (i.e., blood and reference fluid) were set to the same flow rate (i.e., *Q_b_* = *Q_r_*). The flow rate of syringe pump was set to *Q_b_* = 0.025, 0.05, 0.1, 0.2, 0.3, 0.4, 0.5, 0.6, 0.7, 0.8, 0.9, and 1 mL/h. According to the equation for the shear rate in a rectangular blood channel, the shear rate was estimated to be $\dot{\gamma }$ = 679–27,742 s^−1^. Figure 2(C-iii) shows the variations in *µ_b_* with respect to $\dot{\gamma }$. As expected, the plasma viscosity remained constant with respect to the shear rate (*µ_b_* = 1.58 ± 0.04 cP), and the remaining two blood samples exhibited non-Newtonian behavior. Plasma contributed substantially to the increase in blood viscosity compared with 1× PBS. For values greater than *Q_b_* = 0.2 mL/h (i.e., $\dot{\gamma }$ > 4000 s^−1^), the blood viscosity remained constant with respect to the shear rate.

According to the experimental results, the correction factor varied substantially with respect to the channel depth. The blood viscosity increased significantly at higher channel depths. Furthermore, according to the approximation procedure of the nonlinear term in the governing equation, the constant correction factor (CF_0_) was obtained as CF_0_ = 1.183 for a channel depth of *h* = 20 µm. However, the approximation of the nonlinear term did not show a substantial difference when compared with the original nonlinear expression.

### 3.2. Contribution of Channel Depth and Tubing Length to Two Compliance Coefficients

**Figure 3:**
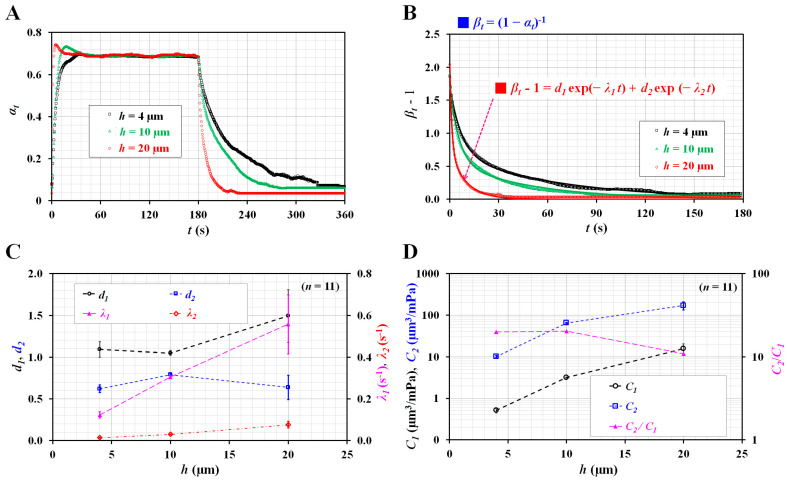
Contribution of channel depth to compliance. (**A**) Temporal variations of *α_t_* with respect to channel depth (*h*) (*h* = 4, 10, and 20 µm). (**B**) Variation of (*β_t_* − 1) with respect to *h*. On the basis of the relationship *β_t_* = (1 − *α_t_*)^−1^, the nonlinear regression formula was assumed as (*β_t_* − 1) = *d*_1_ exp (–*λ*_1_*t*) + *d*_2_ exp (–*λ*_2_*t*). (**C**) Variations of four constants (i.e., *d*_1_, *d*_2_, *λ*_1_, and *λ*_2_) with respect to *h*. (**D**) Variations of *C*_1_, *C*_2_, and *C*_2_/*C*_1_ with respect to *h*.

Before testing the blood, glycerin (30%) and 1× PBS were supplied to the microfluidic channel. As shown in Figure 1(A-i), *Q*_0_ was set to 1 mL/h. Figure 3A shows the temporal variations in α_t_ with respect to channel depth (*h* = 4, 10, and 20 µm). Under transient flow (i.e., *t* > 180 s), the interface decreased substantially at higher channel depths. Using Equation (11), α_t_ was replaced by *β_t_* = (1 − α_t_)^−1^ for calculating the two eigenvalues (i.e., *λ*_1_ and *λ*_2_). Among nonlinear regression models in Matlab, the selected exponential regression model consisted of two exponent terms. To conduct regression analysis with Matlab, on the basis of Equation (12), the regression formula was assumed to be $(\beta_{b}-1)=d_{1}\exp\left(-\lambda_{1}t\right)+d_{2}\exp\left(-\lambda_{2}t\right)$.

As shown in Figure 3B, temporal variations of ($\beta_{b}-1$) were replotted with respect to h. Using a curve-fitting toolbox in Matlab (2022a, Mathworks, Natick, MA, USA), values of four unknown constants (i.e., *d*_1_, *d*_2_, *λ*_1_, and *λ*_2_) were obtained with respect to h. Figure 3C shows the variations of four unknown constants with respect to *h*; a_1_ and a_2_ did not exhibit consistent trends with respect to *h*. However, the two eigenvalues (*λ*_1_ and *λ*_2_) tended to increase substantially with respect to *h*. The two compliance coefficients (*C*_1_ and *C*_2_) were obtained by simultaneously solving Equations (13) and (14). Figure 3D shows the variations in *C*_1_, *C*_2_, and *C*_2_/*C*_1_ with respect to *h*. As expected, *C*_2_ was considerably greater than *C*_1_. However, both *C*_1_ and *C*_2_ tended to increase substantially with respect to h. The ratio of the two compliance coefficients (*C*_2_/*C*_1_) did not exhibit a notable trend with respect to *h*. According to the results, the channel depth was observed to contribute substantially to the increase in the two compliance coefficients.

**Figure 4:**
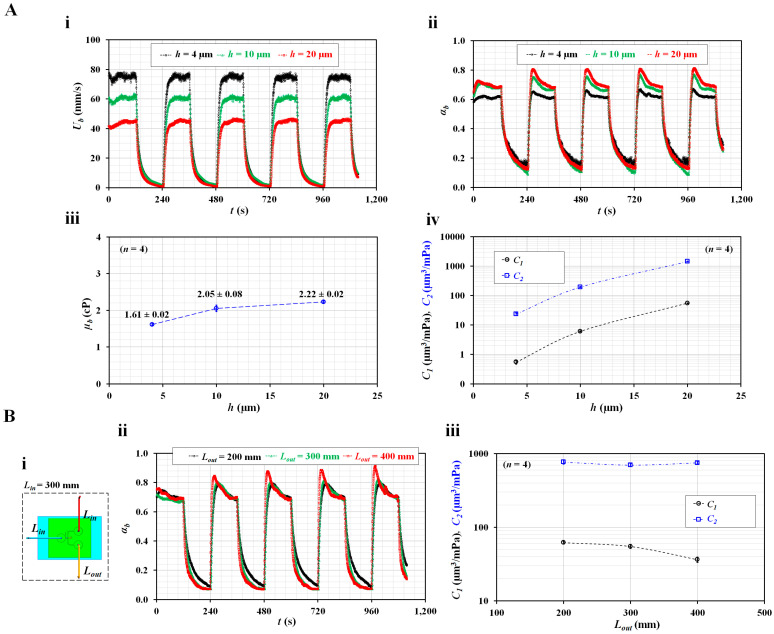
Contributions of channel depth (*h*) and outlet tubing length (*L_out_*) to blood viscosity (*µ_b_*) and two compliances (*C*_1_ and *C*_2_). Blood (Hct = 50%) was prepared by adding normal RBCs into autologous plasma. (**A**) Contribution of channel depth (*h*) to two compliances (*C*_1_ and *C*_2_). (**i**) Temporal variations of blood velocity (*U_b_*) with respect to channel depth (*h*) (*h* = 4, 10, and 20 µm). (**ii**) Temporal variations of interface (*α_b_*) with respect to *h*. (**iii**) Variations of blood viscosity (*µ_b_*) with respect to *h*. (**iv**) Variations of two compliances (*C*_1_ and *C*_2_) with respect to *h*. (**B**) Contribution of outlet tubing length (*L_out_*) to two compliances (*C*_1_ and *C*_2_). (**i**) Schematic of a microfluidic device connected with three kinds of tubing. *L_in_* and *L_out_* represent the length of inlet tubing and length of outlet tubing. The length of inlet tubing was fixed at *L_in_* = 300 mm. The length of outlet tubing set to *L_out_* = 200, 300, and 400 mm. (**ii**) Temporal variations of interface (*α_b_*) with respect to *h*. (**iii**) Variations of two compliances (*C*_1_ and *C*_2_) with respect to *L_out_*.

Instead of glycerin (30%), blood (i.e., normal RBCs in plasma, Hct = 50%) was supplied into the microfluidic channel as the test fluid. The blood viscosity and two compliance coefficients were obtained with respect to the channel depth. To monitor blood flow controlled by a syringe pump, blood velocity (*U_b_*) in the blood channel was obtained using microparticle image velocimetry [31,39,45]. Figure 4(A-i) shows the temporal variations in *U_b_* with respect to *h*; *U_b_* tended to increase substantially at lower channel depths. Here, during the turn-on blood flow interval, *U_b_* did not exhibit consistent variations with respect to channel depth. According to a previous study [46], blood velocity obtained using the micro-PIV technique is influenced by several factors (i.e., hematocrit, diluent, and flow rate). In addition, as shown in Figure 2(C-ii), blood viscosity tended to increase at higher channel depths. Considering that hematocrit contributes to varying blood viscosity, it was estimated that hematocrit might have an influence on the quantification of blood velocity with respect to channel depth.

Figure 4(A-ii) shows the temporal variations in the interface (*α_b_*) with respect to *h*. After turning on a syringe pump, it took a long time to attain steady blood flow. The *α_b_* increased at higher channel depths. Before turning off the syringe pump, at least 30–50 data of interface were selected to calculate blood viscosity. Next, after turning off the syringe pump, two compliance coefficients were obtained by analyzing transient values of *α_b_*. Figure 4(A-iii) shows the variations in *µ_b_* with respect to *h*; blood viscosity tended to increase gradually with respect to *h*. Compared with Figure 2(C-ii), plasma resulted in a larger increase in blood viscosity than 1× PBS. Figure 4(A-iv) shows variations in the two compliance coefficients (*C*_1_ and *C*_2_) with respect to *h*. Both compliance coefficients tended to increase substantially with respect to the channel depth. The results showed that the channel depth substantially increased the blood viscosity and two compliance coefficients.

The outlet tubing connected to the outlet port was expected to have an influence on compliance. A previous study reported that tubing length contributed to the varying compliance coefficient [37]. In subsequent experiments, channel depth was fixed at 20 µm unless otherwise specified. As shown in Figure 4(B-i), the inlet and outlet tubing was connected to the inlet and outlet, respectively, with *L_in_* and *L_out_* representing the lengths of the inlet and outlet tubing, respectively. The length of the inlet tube was fixed at *L_in_* = 300 mm, and that of the outlet tubing was set to *L_out_* = 200, 300, and 400 mm. Blood (RBCs in plasma, Hct = 50%) was supplied in a periodic on/off pattern (*Q*_0_ = 1 mL/h, *T* = 240 s). Figure 4(B-ii) shows the temporal variations in *α_b_* with respect to *h*. At a steady value of *α_b_*, a minor difference in *α_b_* with respect to *L_out_* was observed. The blood viscosity was consistently obtained as *µ_b_* = 2.46 ± 0.07 cP. Using transient variations in *α_b_*, two compliance coefficients were obtained with respect to *L_out_*. As shown in Figure 4(B-iii), *C*_1_ tended to decrease gradually with respect to *L_out_*; however, *C*_2_ remained constant with respect to *L_out_*.

According to the experimental results, the outlet tubing length was found to decrease *C*_1_. However, this did not substantially influence *C*_2_.

### 3.3. Contribution of Hematocrit and Diluted Plasma to Two Compliance Coefficients

To quantify the contribution of blood to the two compliance coefficients, several types of blood were prepared by varying the hematocrit or plasma concentration. Subsequently, blood viscosity and two compliance coefficients were obtained with respect to the hematocrit and plasma concentration.

**Figure 5:**
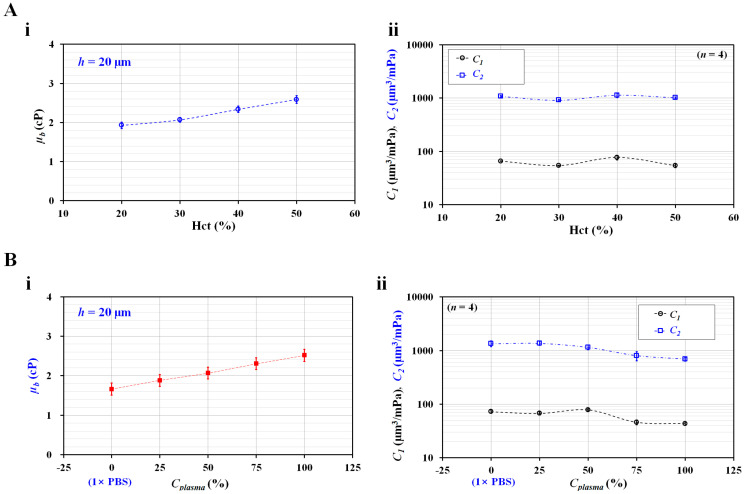
Contributions of hematocrit and diluted plasma to blood viscosity (*µ_b_*) and two compliances (*C*_1_ and *C*_2_). Hematocrit of suspended blood was adjusted by adding normal RBCs into autologous plasma. Autologous plasma was diluted with 1× PBS. Channel depth was fixed at *h* = 20 µm. (**A**) Contributions of hematocrit (Hct = 20%, 30%, 40%, and 50%) to blood viscosity (*µ_b_*) and two compliances (*C*_1_ and *C*_2_). (**i**) Variations of *µ_b_* with respect to Hct. (**ii**) Variations of two compliances (*C*_1_ and *C*_2_) with respect to Hct. (**B**) Contributions of diluted plasma to blood viscosity (*µ_b_*) and two compliances (*C*_1_ and *C*_2_). (**i**) Variations of *µ_b_* with respect to plasma concentration (*C_plasma_*) (*C_plasma_* = 0%, 25%, 50%, 75%, and 100%). *C_plasma_* = 0% and *C_plasma_* = 100% denote pure 1× PBS and autologous plasma, respectively. (**ii**) Variations of two compliances (*C*_1_ and *C*_2_) with respect to *C_plasma_*.

Firstly, to quantify the effect of hematocrit on the two compliance coefficients, the hematocrit of suspended blood was adjusted to Hct = 20%, 30%, 40%, and 50% by adding normal RBCs into autologous plasma. As shown in Figure 2B, because the difference between the nonlinear and approximation terms decreased substantially at higher values of channel depth, the channel depth was set to *h* = 20 µm in subsequent blood tests. Figure 5(A-i) shows the variations in *µ_b_* with respect to Hct. Blood viscosity tended to increase significantly with respect to hematocrit. In addition, as shown in Figure 5(A-ii), the two compliance coefficients were obtained with respect to hematocrit. Both compliance coefficients exhibited a distinctive pattern (fluctuation) with respect to hematocrit. According to the results, the blood viscosity was a better indicator than the compliance coefficient for detecting the contribution of hematocrit in suspended blood. Considering that the compliance coefficient was influenced by hematocrit (or blood viscosity), the hematocrit was fixed at Hct = 50% for consistent measurements.

Secondly, to verify the contribution of plasma concentration to blood viscosity and compliance coefficients, autologous plasma was diluted to *C_plasma_* values of 0%, 25%, 50%, 75%, and 100%; *C_plasma_* = 0% and *C_plasma_* = 100% denote pure 1× PBS and autologous plasma, respectively. Suspended blood (Hct = 50%) was prepared by adding normal RBCs to the diluted plasma. Figure 5(B-i) shows the variations in *µ_b_* with respect to *C_plasma_*. As expected, blood viscosity increased at higher plasma concentrations. Autologous plasma contributed to an increase in the blood viscosity compared with 1× PBS. Figure 5(B-ii) shows the variations in the two compliance coefficients (*C*_1_ and *C*_2_) with respect to *C_plasma_*; *C*_1_ tended to decrease substantially above *C_plasma_* = 50%, and *C*_2_ tended to gradually decrease above *C_plasma_* = 25%. In other words, both compliance coefficients decreased substantially above a specific plasma concentration.

On the basis of these results, blood viscosity and compliance coefficients could be used to detect differences in blood (i.e., hematocrit or diluent). To obtain consistent results, it was necessary to fix the hematocrit concentration. Furthermore, for detecting changes in the hematocrit or diluent, the blood viscosity was a better indicator than the two compliance coefficients.

### 3.4. Detection of Homogenous Hardened RBCs and Heterogeneous RBCs

Furthermore, the present method was employed to detect homogeneous hardened RBCs and heterogeneous RBCs in terms of blood viscosity and two compliance coefficients.

**Figure 6:**
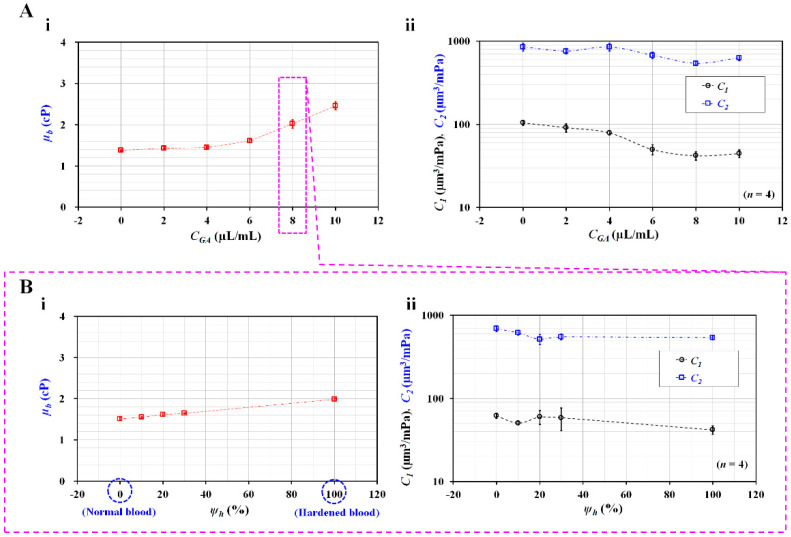
Detection of homogeneous hardened RBCs and heterogeneous hardened RBCs with blood viscosity and two compliances. (**A**) Detection of homogeneous hardened blood composed of RBCs hardened with the same concentrations of GA solution. Normal RBCs were hardened using the specific concentrations of GA solution. After that, hematocrit of blood was adjusted to 50% by adding hardened RBCs into 1× PBS. (**i**) Variations of *µ_b_* with respect to concentrations of GA solution (*C_GA_*) (*C_GA_* = 0, 2, 4, 6, 8, and 10 µL/mL). (**ii**) Variations of two compliances (*C*_1_ and *C*_2_) with respect to *C_GA_*. (**B**) Detection of heterogeneous blood composed of normal blood and hardened blood. Hardened blood was prepared by adding hardened RBCs (*C_GA_* = 8 µL/mL) into PBS solution. Specifically, the volume fraction of hardened blood (*ψ_h_*) was defined as the ratio of hardened blood volume to total blood volume (i.e., *ψ_h_* = *V_h_*/[*V_h_* + *V_n_*], where *V_h_* is the hardened blood volume, and *V_n_* is the normal blood volume). (**i**) Variations of *µ_b_* with respect to *ψ_h_* = 0%, 10%, 20%, 30%, and 100%. (**ii**) Variations of two compliances (*C*_1_ and *C*_2_) with respect to *ψ_h_*.

According to a previous study [40], normal RBCs were rigidified with specific concentrations of GA solution (*C_GA_* = 0, 2, 4, 6, 8, and 10 µL/mL); RBC rigidity increased substantially when normal RBCs were exposed to higher concentrations of the GA solution. Homogeneous hardened blood (Hct = 50%) was prepared by adding homogeneous hardened RBCs to 1× PBS. As shown in Figure 1(A-i), the flow rates of both the fluids were set to *Q*_0_ = 1 mL/h. Figure 6(A-i) shows the variations in *µ_b_* with respect to *C_GA_*; the blood viscosity increased significantly above *C_GA_* = 4 µL/mL. These results indicate that rigidified RBCs contributed to the increased blood viscosity. The blood viscosity had the maximum value at the highest concentration of *C_GA_* (10 µL/mL). Subsequently, two compliance coefficients were obtained with respect to the degree of RBC rigidity (i.e., the concentration of the GA solution). Figure 6(A-ii) shows the variations in the two compliance coefficients (*C*_1_ and *C*_2_) with respect to *C_GA_*; *C*_1_ decreased gradually from *C_GA_* = 0 µL/mL to 8 µL/mL, while *C*_2_ decreased significantly from *C_GA_* = 4 µL/mL to *C_GA_* = 8 µL/mL. *C*_1_ and *C*_2_ remained constant between *C_GA_* = 8 µL/mL and *C_GA_* = 10 µL/mL. These results indicate that RBC rigidity had a maximum value at a concentration of *C_GA_* = 8 µL/mL. The results of the present method are comparable to those of a previous study [40]. Furthermore, the proposed method provided consistent compliance coefficient values (i.e., a smaller standard deviation).

Lastly, the present method was used to detect heterogeneous blood samples. Instead of heterogeneous RBC distributions in the whole blood, this study used the homogeneous normal RBCs and worst deformable RBCs (*C_GA_* = 8 µL/mL) to prepare heterogeneous blood. Each RBC sample was loaded into the same diluent of 1× PBS. Two blood suspensions were partially mixed in terms of a volume fraction of each suspension. The volume fraction of hardened blood (*ψ_h_*) was defined as the ratio of hardened blood volume to total blood volume (i.e., *ψ_h_* = *V_h_*/[*V_h_* + *V_n_*], where *V_h_* is the hardened blood volume, and *V_n_* is the normal blood volume); it was selected as *ψ_h_* = 0%, 10%, 20%, 30%, and 100%. Figure 6(B-i) shows the variations in *µ_b_* with respect to *ψ_h_*; the blood viscosity tended to increase gradually with respect to *ψ_h_*. When the volume fraction of the hardened RBCs increased within a certain amount of blood, the blood viscosity tended to increase. Furthermore, two compliance coefficients were obtained with respect to *ψ_h_*. Figure 6(B-ii) shows the variations in the two compliance coefficients (*C*_1_ and *C*_2_) with respect to *ψ_h_*; *C*_1_ tended to decrease gradually above *ψ_h_* = 20%, while *C*_2_ tended to decrease substantially between *ψ_h_* = 0% and *ψ_h_* = 20%, remaining constant above *ψ_h_* = 20%. The results indicate that blood viscosity and *C*_2_ can be regarded as effective parameters for detecting minor subpopulations in blood.

From the experimental results, it can be concluded that the blood viscosity and two compliance coefficients can be used to effectively detect homogeneous or heterogeneous RBCs.

## 4. Conclusions

In this study, the blood viscosity and compliance coefficients were quantified simultaneously by analyzing the interfacial location of blood flow in a coflowing channel. Two compliance coefficients (i.e., *C*_1_ and *C*_2_) were newly suggested to separate the effect of RBC elasticity within the microfluidic system (i.e., soft PDMS device and flexible tubing). Herein, *C*_1_ denoted the combined effect of the PDMS tubing and PDMS device, while C_2_ represented the elastic effect of RBCs. *C*_1_ was assumed to be much smaller than *C*_2_. Using a discrete fluidic circuit modeling technique, a new governing equation for the microfluidic system was derived and expressed as a second-order nonlinear differential equation. Following approximation procedures, the governing equation became a linear differential equation, and the analytical solution for the governing equation was derived. According to the governing equation, the blood viscosity was obtained under steady blood flow. Next, under transient blood flow, nonlinear regression analysis was conducted to obtain two eigenvalues. Two compliance coefficients were obtained by solving two nonlinear equations. Firstly, according to the linear approximation procedure for the nonlinear term in the governing equation, the constant correction factor (CF_0_) was obtained as CF_0_ = 1.183 for a channel depth of *h* = 20 µm. The linear approximation term was in good agreement with the nonlinear term. Secondly, for glycerin solution (30%) as the test fluid, *C*_2_/*C*_1_ was estimated to be approximately 10.9–20.3 with respect to channel depth (*h* = 4, 10, and 20 µm). The channel depth caused an increase in the two compliance coefficients and blood viscosity. However, the outlet tubing length contributed to a decrease in *C*_1_. Thirdly, for detecting changes in blood (i.e., hematocrit or diluent), blood viscosity was a better indicator than the two compliance coefficients. Lastly, the present method was adopted to detect homogeneously and heterogeneously hardened RBCs. Consequently, the blood viscosity and two compliance coefficients exhibited substantially different trends with respect to the blood suspensions. In conclusion, the proposed method can be used to effectively detect changes in blood or microfluidic systems. The present method was used to detect artificial heterogeneous RBCs, composed of normal RBCs and partially hardened RBCs. However, in a clinical setting, it is necessary to detect subpopulations, deformability, and density in patient blood [47,48]. In future studies, the present method can contribute to detecting the heterogeneity of RBCs in patient blood.
